# Repair effect of the poly (D,L-lactic acid) nanoparticle containing tauroursodeoxycholic acid-eluting stents on endothelial injury after stent implantation

**DOI:** 10.3389/fcvm.2022.1025558

**Published:** 2022-11-08

**Authors:** Jiedong Zhou, Jingfan Weng, Xingxiao Huang, Shimin Sun, Qi Yang, Hui Lin, Jinjin Yang, Hangyuan Guo, Jufang Chi

**Affiliations:** ^1^Department of Cardiology, Shaoxing People’s Hospital (Shaoxing Hospital, Zhejiang University School of Medicine), Shaoxing, China; ^2^Zhejiang Hospital Affiliated to Medical College of Zhejiang University, Hangzhou, China; ^3^Shaoxing University School of Medicine, Shaoxing, China

**Keywords:** ERS, apoptosis, TUDCA, re-endothelialization, PDLLA nanoparticle-eluting stent

## Abstract

**Background:**

Chronic endoplasmic reticulum stress (ERS) plays a crucial role in cardiovascular diseases. Thus, it can be considered a therapeutic target for these diseases. In this study, poly (D,L-lactic acid) (PDLLA) nanoparticle-eluting stents loaded with tauroursodeoxycholic acid (TUDCA), an ER stress inhibitor, was fabricated to assess their ability to reduce endothelial cell apoptosis and promote re-endothelialization after stent implantation.

**Materials and methods:**

PDLLA nanoparticles loaded with TUDCA were prepared *via* the emulsification-solvent evaporation method. The cumulative release rates of TUDCA were measured *in vitro via* high-performance liquid chromatography. The carotid arteries of rabbits were subsequently implanted with stents *in vivo*. The rabbits were then sacrificed after 4 weeks for scanning electron microscopy. Meanwhile, TUDCA concentration in the homogenate of the peripheral blood and distal vascular tissue after stent implantation was measured. The effect of TUDCA on ERS, apoptosis, and human umbilical vein endothelial cell (HUVEC) function was investigated *in vitro* by performing cell migration assay, wound healing assay, cell proliferation assays, endoplasmic reticulum (ER)-specific fluorescence staining, immunofluorescence, and western blotting.

**Results:**

TUDCA nanoparticles were released slowly over 28 days. In addition, TUDCA-eluting stents enhanced re-endothelialization and accelerated the recovery of endotheliocytes *in vivo*. ERS and apoptosis significantly increased in H_2_O_2_-treated HUVECs *in vitro*. Meanwhile, TUDCA reduced apoptosis and improved function by inhibiting ERS in H_2_O_2_-treated HUVECs. Decreased rates of apoptosis and ERS were observed after silencing XBP-1s in H_2_O_2_-treated HUVECs.

**Conclusion:**

TUDCA can inhibit apoptosis and promote re-endothelialization after stent implantation by inhibiting IRE/XBP1s-related ERS. These results indicate the potential therapeutic application of TUDCA as a drug-coated stent.

## Introduction

Currently used drug-eluting stents (DES) reduce the rate of in-stent restenosis. However, these also delay re-endothelialization, which leads to late and very late thrombosis after percutaneous coronary intervention (PCI) (1–4). Endoplasmic reticulum stress (ERS) is a condition in which various stimuli inhibit protein maturation and promote the accumulation of misfolded proteins in organelles. Multiple studies have shown that increased ERS can be observed in damaged cells in many disease models (5–12), and ERS causes the delay in re-endothelialization of the stent segment (13–16).

Tauroursodeoxycholic acid (TUDCA) is a natural hydrophilic bile acid that has been used to dissolve cholesterol stones and is currently known as an ERS inhibitor. TUDCA plays a positive role in a variety of disease models, including hepatobiliary diseases (17–19), diabetes (5, 20), and cancer (8, 9, 21).

In recent years, TUDCA has been shown to delay restenosis after stent implantation by inhibiting vascular smooth muscle cell (VSMC) dedifferentiation (22). In addition, studies have shown that TUDCA can promote endothelial cell proliferation and migration, thereby promoting angiogenesis and repair (23, 24). Therefore, we hypothesized that TUDCA could improve endothelial injury after stent implantation by potentially inhibiting ERS.

In this study, PDLLA nanoparticles containing TUDCA-eluting stents were tested *in vivo* to determine their potential role in promoting re-endothelialization. *In vitro* experiments verified that TUDCA could alleviate ERS to improve endothelial cell apoptosis and promote endothelial function recovery. Our study will help to evaluate the potential use of TUDCA and TUDCA-eluting stents as a new DES.

## Materials and methods

### Preparation of poly (D,L-lactic acid) nanoparticles containing tauroursodeoxycholic acid

Poly (D,L-lactic acid) (PDLLA) nanoparticles loaded with TUDCA were prepared *via* the emulsification-solvent evaporation method. PDLLA nanoparticle containing TUDCA-eluting stents were prepared *via* soaking. Different doses of TUDCA (10 mg, 20 mg, and 40 mg) and PDLLA (polymerization degree of 10 k) were dissolved in dichloromethane (5 mL). They were then mixed completely. Thereafter, 1% polyvinyl alcohol solution was added to the mixture. The mixture was stirred until the dichloromethane was fully volatilized to obtain a mixture of nanoparticles. The emulsion was centrifuged, dispersed ultrasonically, washed three times with distilled water, and freeze-dried to create PDLLA nanoparticles loaded with ursodeoxycholic acid.

### Determination of physicochemical properties of the nanoparticles

The nanoparticles were sprayed with gold, and their surface morphology was observed under a scanning electron microscope (SEM).

The target nanoparticles (50 mg) were dissolved in dichloromethane (1 mL) and acetone in a mixture ratio of 3:7, and the supernatant was discarded after centrifugation. The precipitate was dissolved in phosphate buffer saline (PBS) when the organic solvent was completely volatilized, and its content was analyzed *via* high-performance liquid chromatography (HPLC). Subsequently, entrapment (EE) and loading efficiencies (LE) were calculated. The TUDCA EE was the proportion of the mass of TUDCA in the nanoparticles in the input mass of TUDCA. The equation was as follows:


EE%=massofTUDCAinthenanoparticles/t⁢h⁢e⁢i⁢n⁢p⁢u⁢t⁢m⁢a⁢s⁢s⁢o⁢f⁢T⁢U⁢D⁢C⁢A×100%.

The TUDCA loading efficiency (LE) was calculated as the ratio of the mass of TUDCA in the nanoparticles to the total mass of the nanoparticles. The equation was as follows:


LE%=themassofTUDCAinthenanoparticles/

t⁢h⁢e⁢t⁢o⁢t⁢a⁢l⁢m⁢a⁢s⁢s⁢o⁢f⁢t⁢h⁢e⁢n⁢a⁢n⁢o⁢p⁢a⁢r⁢t⁢i⁢c⁢l⁢e⁢s×100%.

### Determination of the release of tauroursodeoxycholic acid-loaded nanoparticles *in vitro*

Dynamic dialysis was performed to investigate the release of TUDCA-loaded nanoparticles (NPs) *in vitro*. Approximately 10 mg/mL of TUDCA-loaded nanoparticles was dissolved in PBS buffer solution and sealed in a dialysis bag maintained at 37°C. At specific time points (1 h, 2 d, 3 d, 4 d, 7 d, 10 d, 14 d, 21 d, 28 d), 1 mL of the release medium was taken to measure the drug content. At the same time, an equal volume of PBS was added to the dialysis bag buffer. The TUDCA content was detected using HPLC. The cumulative release rate was calculated, and a drug release curve was drawn.


$$CumulativeReleaseRate\left(\%\right)=\frac{c_{t}\times V_{o}+\sum_{n=1}^{t-1}c\times V}{M_{0}}\times 100\%$$

c_*t*_: content of TUDCA in each sample; V_0_: release medium volume; V: sampling volume; M_0_: total amount of TUDCA in the release medium.

### MTT assay

A total of 5 × 10^4^/mL HUVECs were added to a 96-well plate. Precisely, 100 μl of HUVECs was added per well. The 96-well plate was then placed in an incubator for 24 h. The experiment was divided into three groups: control group (blank DMEM culture medium), PDLLA group (leach liquor of blank-loaded nanoparticles), and PDLLA+TUDCA (leach liquor of TUDCA-loaded nanoparticles).

Four replicate holes were created for each group. Approximately 100 μL of the intervention solution was added to each well and was incubated for 24 h. Afterward, 10 μL of 5 mg/mL MTT solution was added to each well and incubated for 4 h. Around 100 μL of DMSO was added to each well, which was then shaken for 10 min to dissolve the blue-purple crystals. A microplate reader was used to measure the absorbance of each well at 570 nm and to calculate drug toxicity.

### Preparation of poly (D,L-lactic acid) nanoparticle containing tauroursodeoxycholic acid-eluting stents

By adopting the soaking process using dichloromethane as the solvent and PDLLA as the carrier, five target stents were made after two soaking and drying treatments. As a result, five target stents were made.

### Tauroursodeoxycholic acid, nitric oxide, and endothelin-1 concentration detection *in vivo*

Before the rabbits were sacrificed, 4 mL of peripheral blood was drawn, and the serum was collected after centrifugation. Simultaneously, 1-cm blood vessels above and below the covered area of the PDLLA nanoparticle containing the TUDCA-eluting stent were removed to prepare a tissue homogenate. The TUDCA content in the vascular tissue homogenate around the stent and peripheral blood serum was detected *via* HPLC. The concentration of ET-1 and NO in serum were detected using ET-1 ELISA kit (Abcam, ab133030) and NO Kit (Beyotime, Haimen, China).

### Endothelial injury rabbit model stent implantation

The protocols used for all animal studies were approved by the Zhejiang University Animal Policy and Welfare Committee and were in compliance with the NIH guidelines (Guide for the Care and Use of Laboratory Animals).

New Zealand White Rabbits (2.0–3.0 kg, Dashi Juxin Rabbit Farms, Xinchang Country, Zhejiang Province, China) were randomly divided into four groups: Sham (*n* = 5, balloon; Multi-Link Vision, Abbott Vascular, Chicago, IL, USA), BMS (bare metal stent) (*n* = 5; BMS, Multi-Link Vision, Abbott Vascular, Chicago, IL, USA), BMS+oral TUDCA (*n* = 5; BMS, Multi-Link Vision, Abbott Vascular, Chicago, IL, USA; TUDCA, Taurolite, Bruschettini S.R.L, Genova, Italy), and TUDCA-eluting stent+oral TUDCA, (*n* = 5; BMS, Mu006Cti-Link Vision, Abbott Vascular, Chicago, IL, USA).

The rabbits were anesthetized *via* inhalation of sevoflurane. The BMS and TUDCA-eluting stents were fully attached to the balloon and then sent into the left carotid artery. After that, the stents were inflated *via* balloon pumping to 8F, and implanted. In the sham group, the balloon was sent into the left carotid artery, pumped to 5F, and pulled back and forth 3–4 times in the lumen to damage the intima (22, 25, 26). After the operation, 40 mg aspirin and 70 mg ticagrelor were administered to all rabbits daily for 7 days. The BMS+TUDCA and TUDCA-eluting stent+TUDCA groups were administered with 100 mg/kg oral TUDCA for 28 days.

### Euthanasia and fixation

At the end of the 28 days, the rabbits underwent overnight fasting. They were then anesthetized and the rabbits’ neck skin was incised, and the left carotid artery was obtained and perfused at 80 mmHg, dropping from a height of 2 m with Ringer’s lactate until the perfusate from the jugular vein was clear.

### Scanning electron microscopy

The stented arterial segments were fixed in glutaraldehyde solution, longitudinally bisected to expose the luminal surface, and photographed. SEM was used to determine the cell coverage on the stent surface at incremental magnifications of × 500 and × 2,500.

### Histological evaluation

Stented sections were stained with hematoxylin and eosin (HE). Changes in endothelial coverage of the lumen were observed after HE staining.

### Cell culture and treatment

HUVECs were obtained from the Cell Bank of Chinese Academy of Sciences (Shanghai, China) and cultured in DMEM (Sigma, St Louis, MO, USA) containing 10% fetal bovine serum (Gibco, Grand Island, NY, USA) at 37°C in a humidified atmosphere with 5% CO_2_. HUVECs were cultured to 70–80% confluence (passage numbers: 3–5) before using them in the experiments. Groups of experiments are as follows: Control group: (DMEM + 10% FBS), TUDCA group (TUDCA 1000 uM), H_2_O_2_ group (H_2_O_2_ 300 μM), H_2_O_2_+TUDCA group (H_2_O_2_ 300 μM +TUDCA 1000 uM). HUVECs were treated with H_2_O_2_ (300 μM) for 3 h to establish a cell injury model, and TUDCA co-incubated for 48 h.

To knockdown XBP1 in HUVECs, XBP1 small interfering RNA (XBP1-siRNA) lentivirus and control siRNA lentivirus oligonucleotides were synthesized by Genechem (Shanghai, China). A single dose of 10 MOI of control small interfering ribonucleic acid (siRNA) was administered to the cells at 60% confluency, according to the manufacturer’s instructions. Knockdown efficiencies were tested *via* western blotting 48 h after siRNA transfection. XBP1-siRNA HUVECs were treated with the same pattern as described above.

### CCK-8 assay

HUVECs between passages 3 and 5 were serum-starved overnight, followed by digestion of the cell monolayer using 0.25% trypsin. DMEM containing 10% FBS was added to the cell suspension, and the cells were counted. Each well was seeded with 5 × 10^4^ cells (200 μL) in a 96-well culture plate.

In the control group, cells were cultured without treatment. In the H_2_O_2_ group, cells were treated with H_2_O_2_ (300 μM for 3 h and then cultured in DMEM with 10% FBS). In the experimental group, cells were pretreated with H_2_O_2_ (300 μM), then cultured in TUDCA (10, 100, 1,000 μM), respectively for 24, 48, and 72 h.

Both control and experimental groups were cultured at 37°C with 5% CO_2_. After treatment, CCK-8 solution was added, and the cells were incubated for 4 h. Absorbance at 455 nm was determined using a microplate reader (Bio-Rad, Hercules, CA, USA).

### Annexin-V/PI assay

Annexin-V/PI assays were performed using apoptosis assay kit (Roche, Basel, Switzerland) in accordance with the manufacturer’s instructions. Briefly, HUVECs were cultured in 6 well plates at a density of 2.0 × 10^5^ cells/well and incubated in DMEM supplemented with 10% FBS. One day later, the cells were washed and incubated in medium with various concentrations of H_2_O_2_ (100, 300, 500 uM) for 2 h. cells were harvested and washed three times with PBS. Subsequently, the suspensions were transferred to 1.5-mL tubes, and 5 μL of Annexin V and 10 μL of PI solution were added. The cells were incubated in the dark at room temperature for 20 min, and cell counting was performed using a FACSCalibur (Beckman coulter, Brea, CA, USA).

### Wound healing assay

HUVECs were cultured to 80% confluence, serum-starved, and treated with hydroxyurea overnight for synchronization and growth inhibition. A wound was created in the HUVEC monolayer using a sterile 100 μL pipette tip. PBS was used to flush the 6-well plate and wash away cell debris. The cells were analyzed at 0 and 24 h post-wounding using a Nikon microscope (Nikon Corporation, Tokyo, Japan). The ratio of the cell recovery area to the entire wound area was calculated to evaluate cell migration.

### Apoptosis assay

An *in situ* cell death detection kit, TUNEL (Roche Co. Ltd., Basel, Switzerland), was used to detect DNA fragmentation in the individual cells. HUVECs were incubated with the TdT-mediated dUTP nick end labeling (TUNEL) reaction mixture at 37°C for 1 h in the dark after fixation with 4% paraformaldehyde and permeation with 0.2% Triton. After the nuclei were counterstained with 0.1 μg/mL DAPI (Sigma-Aldrich; St Louis, MO, USA; D9564) for 3 min, the number of TUNEL-positive cells was counted using a Leica microscope at 200 × magnification.

### Immunofluorescence

HUVECs were used in immunofluorescence assays to visualize the expression of GRP78 and XBP-1s. Briefly, the HUVEC monolayer was fixed at room temperature for 15 min, followed by incubation in a blocking buffer (4% BSA) for 30 min. Subsequently, immunostaining was performed using a primary antibody. Nuclei were counterstained with 0.1 μg/mL DAPI (Sigma-Aldrich; St Louis, MO, USA; D9564). Images were captured using a Nikon Eclipse Ti-U fluorescence microscope at × 200 magnification. The specific antibodies used in this study were GRP78 (ab108615; Abcam, Cambridge, England) and BCL-2 (ab692; Abcam, Cambridge, England).

### Endoplasmic reticulum morphology capture

After the intervention of cells described above, cells were incubated with ER fluorescent probes (Beyotime, Haimen, China) for 15 min at 37°C and nuclei were counterstained with hoechst, the ER image is capture using a lase confocal microscope (Leica Stellaris, Wezler, Germany).

### Western blotting

HUVECs were grown in DMEM supplemented with 10% FBS. Before processing the cells for western blotting, the culture medium was removed and the cells were washed three times with PBS. HUVECs were lysed with a lysis buffer (Beyotime, Haimen, China) on ice for 30 min. Cell lysates were centrifuged at 12,000 × *g* for 15 min at 4°C. Equal amounts of soluble protein were separated by 12% SDS-PAGE, and the protein bands were electro-transferred onto polyvinylidene fluoride membranes. The membranes were blocked with 5% skim milk, followed by overnight incubation with the primary antibody. Secondary antibodies conjugated to horseradish peroxidase were used in the subsequent experiments. An enhanced chemiluminescence (ECL) detection kit was used to visualize the target proteins and internal control. Protein bands were obtained by autoradiography and analyzed *via* Quantity One 4.4 (Bio-Rad, Hercules, CA, USA). The specific antibodies used in this study were as follows: anti-IRE1α (ab48187, Abcam, Cambridge, England), anti-XBP1s (#12782, CST, Danvers, MA, USA), anti-CHOP (#2895, CST, Danvers, MA, USA), anti-BAX (#89477, CST, Danvers, MA, USA), anti-BCL2 (ab692, Abcam, Cambridge, England), anti-GAPDH (#5174, Danvers, MA, USA), HRP-conjugated goat anti-rabbit (Beyotime, Haimen, China, A0208) and HRP-conjugated goat anti-mouse (Beyotime, Haimen, China, A0216).

### Real-time reverse-transcription PCR

Total RNA was isolated from hearts tissues using TRIzol reagent (Invitrogen, Carlsbad, CA, USA) following the manufacturer’s protocol. RNA was re-verse-transcribed with oligo (dT) primers and real-time PCR was conducted on an ABI 7300 RT-PCR Detection System (Applied Biosystems, Foster, CA, USA) with gene-specific primers in the presence of SYBR Premix Ex Taq (TaKaRa, Dalian, China). RT-qPCR was performed in tripli-cate. Relative fold changes in the expression of the target gene were determined using the 2^–ΔΔCT^ method. The primer sequences are provided in Supplementary material.

### Statistical analysis

Each *in vitro* experiment was repeated at least thrice using a fresh batch of cells for each experiment. Statistical Package for the Social Sciences Statistical Package for the Social Sciences version 19.0 statistical software (SPSS Inc., Chicago, IL, USA) was used to perform the statistical analyses. Data are expressed as mean ± standard deviation (SD). Multiple group comparisons were performed using two-way ANOVAs, followed by Bonferroni *post-hoc* tests. The unpaired Student’s *t*-test was used for two-group comparisons. Statistical significance was set at *P* < 0.05.

## Results

### Preparation and physicochemical properties of poly (D,L-lactic acid) nanoparticle containing tauroursodeoxycholic acid-eluting stents

**FIGURE 1:**
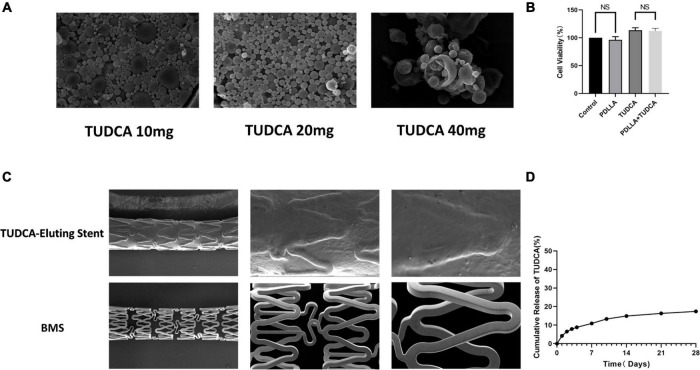
Preparation and physicochemical properties of poly (D,L-lactic acid) (PDLLA) nanoparticle containing tauroursodeoxycholic acid (TUDCA)-eluting stents. **(A)** Scanning electron microscope (SEM) of PDLLA nanoparticle containing TUDCA (10/20/40 mg). **(B)** MTT assay was used to determinate cytotoxicity of the target particles. (NS, not statistically significant, *n* = 4). **(C)** SEM of PDLLA nanoparticle containing TUDCA-eluting stents and BMS. **(D)** The cumulative release rate of TUDCA-loaded nanoparticles was tested *in vitro*.

Different doses of TUDCA (10, 20, and 40 mg) were mixed with PDLLA (polymerization degree of 10 k) to create PDLLA nanoparticles containing TUDCA. We selected the best particles according to their stability and uniformity using SEM (Figure 1A) and determined the collocation of 20 mg TUDCA and 10 k PDLLA. The drug loading and encapsulation rates were determined by HPLC to be 6.65 and 35.4%, respectively. Simultaneously, we tested the cytotoxicity of the target particles using the MTT assay (Figure 1B). As shown in Figure 1B, PDLLA nanoparticles containing TUDCA did not damage HUVECs. In addition, the cumulative release rate of TUDCA-loaded nanoparticles was tested *in vitro*. The results showed that the drug release gradually leveled off after 2 weeks (Figure 1D).

Poly (D,L-lactic acid) (PDLLA) nanoparticles containing TUDCA-eluting stents were prepared by an immersion process and photographed using SEM. The stent was evenly wrapped by particles, and the stent surface was smooth (Figure 1C).

### Tauroursodeoxycholic acid promoted re-endothelialization and inhibited restenosis after stent implantation *in vivo*

**FIGURE 2:**
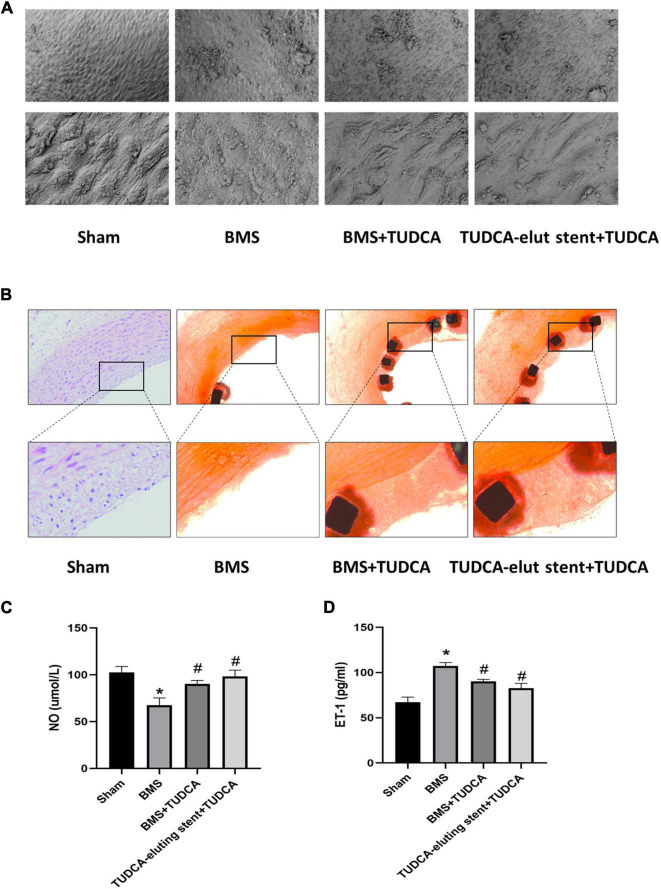
Tauroursodeoxycholic acid (TUDCA) promotes re-endothelialization and inhibits restenosis after stent implantation *in vivo*. **(A)** The vascular surfaces of four groups (Shame, BMS, BMS+TUDCA, TUDCA eluting stent+TUDCA) were covered with endothelial cells. **(B)** Representative histological images of Shame, BMS, BMS+TUDCA, TUDCA eluting stent+TUDCA (HE staining). The framed part is the repaired intravascular surface. **(C,D)** The contents of ET-1 and NO in serum. (*Compared with Sham, #compared with BMS).

Four weeks after the operation, we removed the blood vessel with or without the stent, cut off the vessel at the distal end, and selected the parts with high endothelial coverage for SEM to observe the cell morphology at 500X and 2500X, respectively. As shown in Figure 2A, Compared with the sham, BMS+TUDCA, and TUDCA-eluting stent+TUDCA groups, the coverage density of endothelial cells (ECs) in the BMS group was lower, the cell morphology was shriveled, and a small amount of red blood cells, platelets, and fibrin adhesion was observed. The ECs in the BMS+TUDCA and TUDCA-eluting stent+TUDCA groups were densely covered and full in cell morphology. As shown in Figure 2B, HE staining results of the cross-section of blood vessels showed that compared with the endothelial coverage of the BMS group, that of the BMS+TUDCA and TUDCA-eluting stent+TUDCA groups was significantly improved and closer to that of the sham group. This suggests that TUDCA can improve re-endothelialization, endothelial cell morphology, and wound repair after stent implantation by oral administration or sustained release of nanoparticles or oral administration. The content of ET-1 and NO in serum also changed in each group. Compared with the sham group, implantation of BMS caused NO down-regulation while ET-1 up-regulation, and the imbalance of NO/ET-1 ratio suggested endothelial dysfunction. Application of TUDCA, on the other hand, up-regulated NO and reduced ET-1 serum content, indicating a recovery of vascular endothelial function (show in Figures 2C,D). In addition, we tested the TUDCA concentration *via* HPLC in the homogenates of the peripheral vascular tissue and vascular tissue around stent implantation. Unfortunately, TUDCA was not detected probably because it was below the detection threshold.

### Tauroursodeoxycholic acid promote the proliferation and migration of HUVECs

**FIGURE 3:**
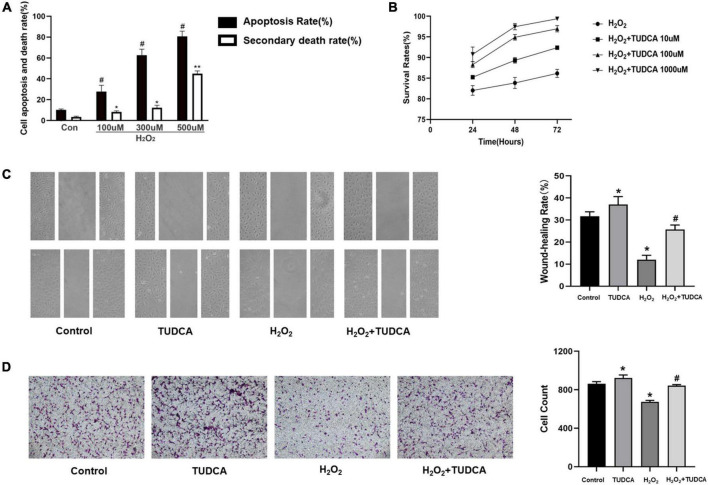
Tauroursodeoxycholic acid (TUDCA) promotes HUVECs proliferation and migration *in vitro*. **(A)** The levels of apoptosis (apoptosis rate) and necrosis (secondary death rate) after exposure to H_2_O_2_ at the indicated doses. Values represent the percentage of cells undergoing each form of death and are presented as mean ± SD; #*p* < 0.05, *p < 0.05, **p < 0.01 compared with normal HUVECs. **(B)** CCK-8 assay was used to assess the cell survival rate of HUVECs. TUDCA improved the survival rate of HUVEC with time and concentration dependence. **(C)** TUDCA promotes HUVEC migrating after H_2_O_2_ induced HUVEC damage in wound-healing assay. (*P < 0.05 vs. control group, *n* = 3; #*P* < 0.05 vs. H_2_O_2_ group, *n* = 3). **(D)** TUDCA promotes HUVEC migrating after H_2_O_2_ induced HUVEC damage in cell migration assay. (*P < 0.05 vs. control group, *n* = 3; #*P* < 0.05 vs. H_2_O_2_ group, *n* = 3).

We measured the apoptosis rate and the mortality after co-incubation with different concentration gradients of H_2_O_2_ for 3 h, and finally we selected 300 umol, which mainly caused apoptosis rather than necrosis, for subsequent apoptosis-related studies (Figure 3A). Then, HUVECs were stimulated with 300 umol/L H_2_O_2_ and treated with 10, 100, or 1,000 μmol/TUDCA for 24, 48, or 72 h, respectively. TUDCA reduced the damage induced by H_2_O_2_ and promoted the proliferation of HUVECs in the cell proliferation assay (Figure 3B). Remission was time-and concentration-dependent. We selected 1,000 uM of TUDCA treated for 48 h as the intervention condition for subsequent *in vitro* modeling. In addition, TUDCA promoted HUVECs migrations after the addition of H_2_O_2_ in cell migration and wound healing assays (Figures 3C,D). These results suggest that TUDCA may promote the proliferation and migration of HUVECs.

### Tauroursodeoxycholic acid relieve H_2_O_2_ induced endoplasmic reticulum stress in HUVECs

**FIGURE 4:**
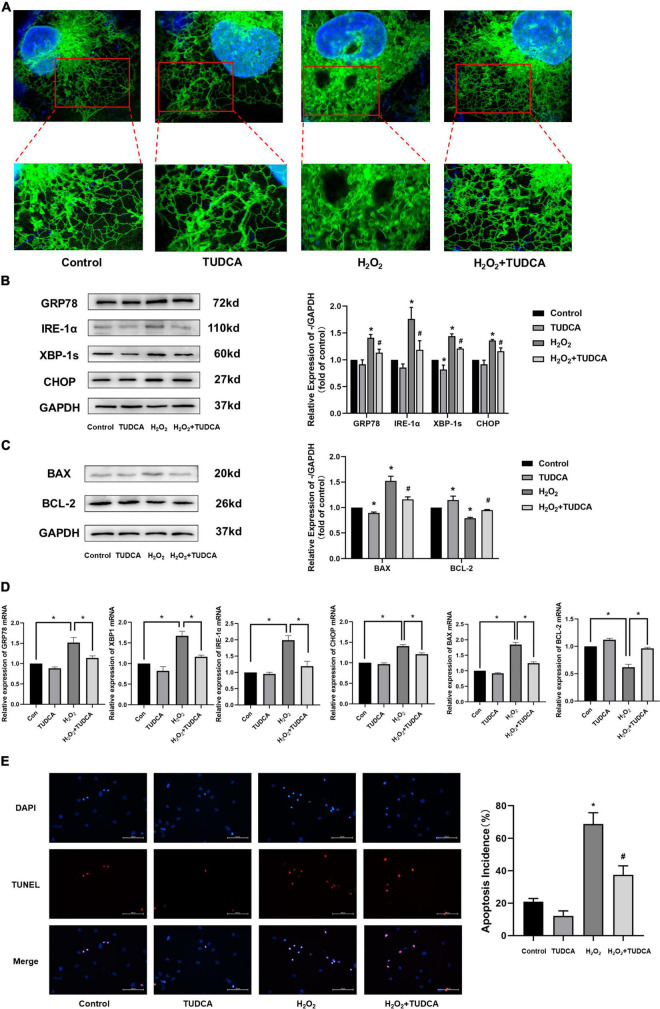
Tauroursodeoxycholic acid (TUDCA) relieves endoplasmic reticulum stress (ERS) and reduces level of apoptosis in HUVECs. **(A)** Representative diagram of ER tracker, blue represents the nucleus and green represents the ER. **(B)** Western blotting was employed to quantitate the expression levels of ER stress marker GRP78, IRE-1α, XBP-1s, and CHOP. (*P < 0.05 vs. control group, *n* = 3; #*P* < 0.05 vs. H_2_O_2_ group, *n* = 3). **(C)** Western blotting was employed to quantitate the expression levels of BAX, BCL-2. (*P < 0.05 vs. control group, *n* = 3; #*P* < 0.05 vs. H_2_O_2_ group, *n* = 3). **(D)** mRNA expression of GRP78, XBP-1s, IRE-1α, CHOP, BAX, BCL-2. **(E)** Effects of TUDCA on H_2_O_2_-induced apoptosis of HUVECs, as detected by TUNEL apoptosis detection. (*P < 0.05 vs. control group, *n* = 3; #*P* < 0.05 vs. H_2_O_2_ group, *n* = 3).

HUVECs were stimulated with 300 μmol/L H_2_O_2_ for 3 h, followed by treatment with TUDCA (1,000 umol/L) for 48 h, and processed for ER fluorescent labeling, immunofluorescence, and western blotting. As shown in Figure 4A, After H_2_O_2_ treatment, the morphology of HUVECs changed significantly; they were mainly shrinking and spreading. The internal structure was disordered. The ER was swollen. In contrast, after TUDCA treatment, the cell structure remained normal, while the ER swelling recovered. These results indicate that HUVECs treated with H_2_O_2_ were damaged, while the ER morphology changed. Contrastingly, TUDCA may protect cells and reduce or even reverse the damage caused by H_2_O_2_.

We measured the levels of the ERS markers, GRP78, IRE-1α, XBP-1s, and CHOP, *via* western blotting. As shown in Figure 4B, HUVECs showed increased GRP78, IRE-1α, XBP-1s, and CHOP protein expression levels in the H_2_O_2_ group compared with those in the control group. Similar results were also observed at the mRNA level (Figure 4D). This indicated that H_2_O_2_ induced ERS in HUVECs. In addition, the proteins expression levels were reduced in HUVECs treated with TUDCA. These results indicated that TUDCA might relieve H_2_O_2_ induced ERS in HUVECs.

Implantation of a stent, a metal foreign body, can cause long-term damage to the endothelium and induce apoptosis of HUVECs, which delay the process of re-endothelialization. Therefore, it is important to inhibit the apoptosis of ECs to promote re-endothelialization. In this study, the expression levels of the apoptosis marker, BAX, and the apoptosis inhibitory protein, Bcl-2, were determined *via* western blotting. The results suggest that TUDCA reduced the level of apoptosis induced by H_2_O_2_ (Figure 4C). Similar results were observed in the TUNEL apoptosis detection (Figure 4E).

### Tauroursodeoxycholic acid reduced apoptosis by inhibiting the IRE/XBP-1s pathway *in vitro*

**FIGURE 5:**
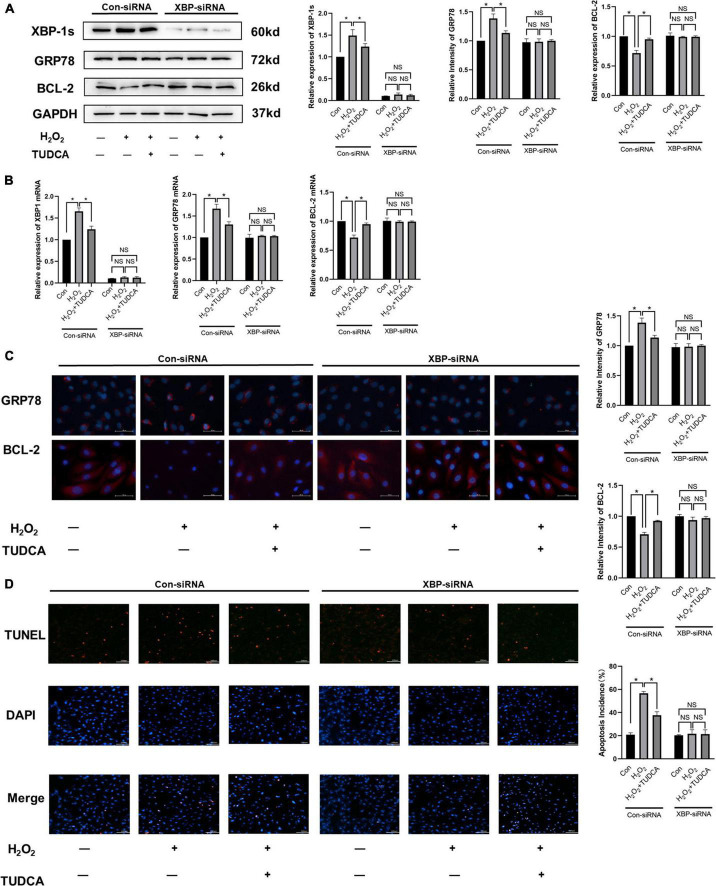
Tauroursodeoxycholic acid (TUDCA) reduced apoptosis by inhibiting the IRE/XBP-1 pathway *in vitro*. **(A)** Western blotting was used to determine the expression levels of XBP-1s, GRP78, BCL-2. (*P < 0.05 *n* = 3). **(B)** mRNA expression of XBP-1s, GRP78, BCL-2. **(C)** Immunofluorescence was used to determine the expression levels of GRP78, BCL-2. (*P < 0.05, *n* = 3). **(D)** Effects of TUDCA and XBP1s in H_2_O_2_-induced apoptosis of HUVECs, as detected by TUNEL apoptosis detection. (*P < 0.05, NS: not statistically significant, *n* = 3).

To further explore the mechanism by which ERS was alleviated by TUDCA in HUVECs, we silenced XBP-1s through lentiviral transfection and observed the changes in the level of apoptosis. Western blotting (Figure 5A) and immunofluorescence (Figure 5C) results revealed that after XBP-1s silencing, XBP-1s and GRP78 expression level decreased. In addition, ERS induced by H_2_O_2_ was reduced, this result was similar to that noted in the control group. We also performed immunofluorescence and western blotting detection of BCL-2, it was then found that TUDCA’s effect of reducing apoptosis disappeared after XBP1s silencing, similar results were observed in the mRNA expression (Figure 5B) and TUNEL apoptosis detection (Figure 5D). The data were further analyzed by two-way ANOVA. The results showed that the main effects of XBP1s and TUDCA had significant effects on ERS and apoptosis rate (*p* < 0.05). Besides, the interaction between XBP1s and TUDCA was statistically significant (*p* < 0.05). These results suggest that XBP-1s plays a major role in ERS induced by H_2_O_2_, and TUDCA reduced apoptosis by inhibiting the IRE/XBP-1s pathway.

## Discussion

In this study, TUDCA reduced HUVEC apoptosis and improved migration by inhibiting ERS, thus promoting the proliferation of HUVECs. PDLLA nanoparticles containing TUDCA slowly released TUDCA. Compared with the BMS group, the TUDCA stent promoted re-endothelialization after stent implantation.

In recent years, PCI with DES has been the mainstream treatment for coronary atherosclerotic heart disease to support blood vessels and reconstruct blood supply. DES contain anti-proliferative drugs such as rapamycin, paclitaxel, and everolimus. DES inhibits VSMC migration, differentiation, proliferation, and the generation of collagen fibers to reduce the incidence of in-stent restenosis (ISR) (1, 22, 27–29). While anti-proliferative drugs resist ISR, the proliferation of ECs is inhibited, and the process of re-endothelialization is delayed. These are the basis of late stent thrombosis and are closely related to endothelial dysfunction (2, 3, 30–32). The additional effects of the eluting polymers are also related to HUVEC damage (4, 33–35).

Endoplasmic reticulum stress (ERS) is a state in which various stimuli, including genetic and environmental insults, lead to impaired protein maturation, resulting in the accumulation of misfolded proteins in organelles. Therefore, the unfolded protein response (UPR) is activated to restore ER homeostasis in three different ways (36, 37): (a) by upregulating the transcription of chaperones, (b) by attenuating translation, and (c) by activating ER-associated degradation. However, UPR plays a dual role in the ERS response. In the UPR adaptive phase, the UPR may alleviate the negative effects of ERS and inhibit apoptosis to a certain extent. However, under long-term and high levels of ERS, the UPR initiates the apoptotic program (38, 39). The IRE1-XBP1 pathway is a conserved and complex signaling pathway in the UPR. XBP1 expression is increased in the areas of blood vessels that have been damaged and inflamed, including atherosclerotic (15, 40–42), vasculitis-affected (43) or stent-implanted vessels (22). As a foreign body, stents implanted into the blood vessels cause long-term and high levels of ERS in ECs, leading to cell apoptosis (22). In this study, we selected the IRE/XBP1-s pathway, the most conservative signaling branch of the UPR, and explored the effect of XBP-1s and its relationship with apoptosis in HUVECs under ERS by silencing XBP-1s *in vitro*. The results showed that TUDCA improved ERS-induced apoptosis through the IRE/XBP-1 signaling pathway.

Tauroursodeoxycholic acid (TUDCA) is a taurine-binding ursodeoxycholic acid. In multiple disease models, TUDCA delays disease progression by alleviating chronic ERS, including cardiovascular diseases (17–19, 37, 44–48), metabolic diseases (5, 20, 49), and neurodegenerative diseases (11, 12, 50–55). Several studies have shown that TUDCA could protect cells through the following three pathways: (1) by reducing ERS and stabilizing the UPR; (2) by activating the pro-survival pathway to inhibit apoptosis; and (3) by reducing inflammation. Moreover, TUDCA has been confirmed to inhibit the superficial transformation of VSMCs, thus inhibiting restenosis after stent implantation. These findings suggest that combining anti-ERS therapies with drug-eluting stent technology may promote the re-endothelialization process after PCI.

This study has some limitations that need to be considered. In this experiment, the intervention times *in vivo* and *in vitro* were short. The determination of the cumulative release rate of TUDCA and the *in vivo* stent intervention period need to be followed *via* extended testing or intervention time to observe the long-term effect of TUDCA. In the *in vivo* experiment, we also detected the drug content in the vascular tissue at the implantation site of the TUDCA-eluting stent and the TUDCA content in the blood vessels in the peripheral tissue, both of which were not detected, which could not prove the key role of TUDCA in the implantation site. In addition, since our tissue section was a hard tissue section containing metal scaffold, which reached a thickness of 100 μm, immunostaining was not possible, which deprived us of the opportunity to perform some research. We aim to find a better detection method in a future study. In addition, studies have shown that XBP-1s can negatively inhibit ERS (56, 57). However, in this experiment, ERS and apoptosis improved after XBP-1s silencing. We hypothesized that apoptosis induced by the chronic UPR response was reduced by XBP-1s silencing or that XBP-1s might lead to apoptosis by inducing an inflammatory response or abnormal metabolism of fats (7, 58–60). XBP1 is one of the important proteins that mediate the occurrence of ERS, the effect of XBP1 on cells depends mainly on the strength of the injury and the degree of activation of XBP1. When the mice are pretreatment with small pre-stimulations such as low-frequency electrical stimulation, the low-level expression of XBP1 is induced then the body is provided with adaptive capacity, which is a protective measure, so that the less cell necrosisbody in body during subsequent severe damages such as hypoxia-reperfusion injury (61). However, if the cells suffer from strong oxidative stress, resulting in high expression of XBP1, it will lead to severe ERS and induce cell necrosis. This shows the difference between chronic and short-term UPR, or the difference between the low level XBP1 and the high level XBP1; however, more studies are warranted to understand the boundary between the two. Go further, in view of the fact that low-level induction of XBP1 seems to enable the body to have some adaptive ability to cope with the subsequent huge attack, before arterial stent implantation, can we perform some pretreatment to induce low-level expression of XBP1, so that endothelial cells have a certain adaptive ability, and thus less apoptosis of endothelial cells may occur after stent implantation? This is our next research goal.

As interventional therapy has become the mainstream treatment method of reperfusion therapy, the excessive proliferation of VSMC and the delay of re-endothelialization have become the main problems to need to be overcome. However, TUDCA reduced intracellular restenosis caused by excessive proliferation of VSMCs by inhibiting the dedifferentiation of VSMCs, which was confirmed in Luo’s study (22). The present study further demonstrated that TUDCA improved ERS-induced apoptosis of ECs, thereby promoting re-endothelialization. Therefore, TUDCA has achieved a good balance between improving restenosis and promoting re-endothelialization.

## Data availability statement

The raw data supporting the conclusions of this article will be made available by the authors, without undue reservation.

## Ethics statement

The animal study was reviewed and approved by Experimental Animal Ethics Committee of Shaoxing People’s Hospital. Written informed consent was obtained from the owners for the participation of their animals in this study.

## Author contributions

JW was responsible for manuscript writing. JZ and XH were responsible for the project implementation. SS, QY, HL, and JY were responsible for the proofreading of manuscript. HG and JC were responsible for the design of the project. All authors contributed to the article and approved the submitted version.
