# Supplementary material for: Repair effect of the poly (D,L-lactic acid) nanoparticle containing tauroursodeoxycholic acid-eluting stents on endothelial injury after stent implantation
Source: Front Cardiovasc Med. 2022 Nov 8;9:1025558. doi: 10.3389/fcvm.2022.1025558 (PMC9678935; doi:10.3389/fcvm.2022.1025558)
Supplement: Supplementary file 1 [file Table_1.docx]

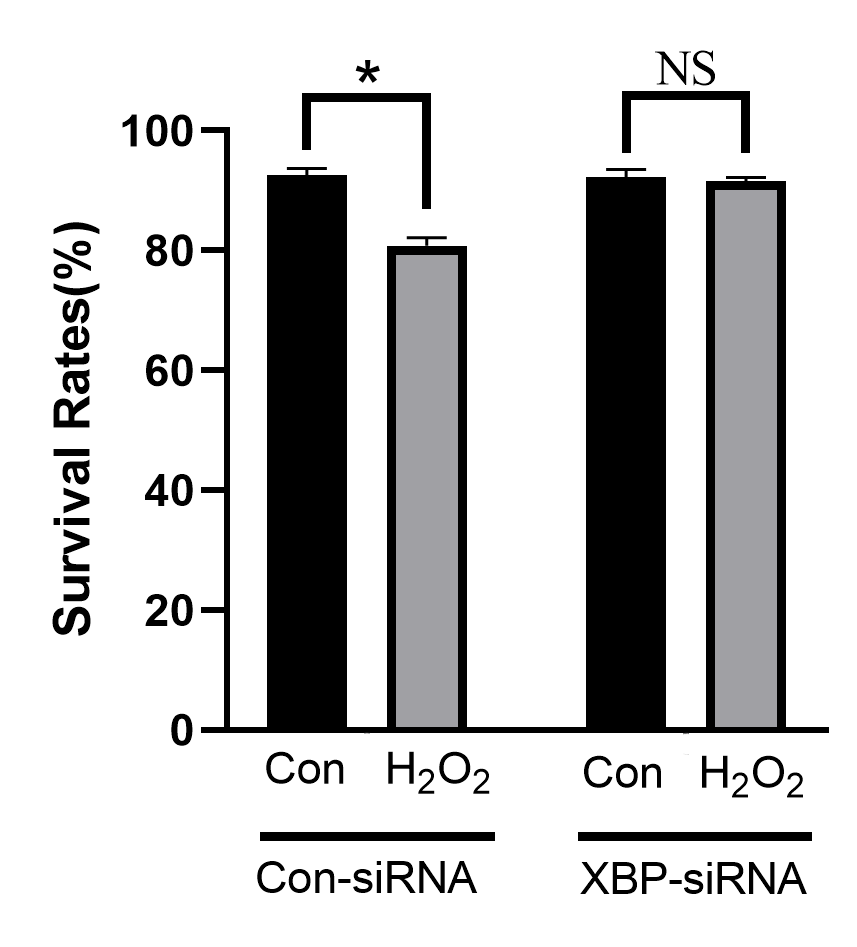


Figure I: Effect of XBP1s on cell viability. (*p<0.05, NS: not statistically significant)

**Primer sequence:**

**GAPDH:**

(F) CAAGGCTGTGGGCAAGGTCATC

(R) GTGTCGCTGTTGAAGTCAGAGGAG

**XBP1s**

(F) CTGAGTCCGCAGCAGGTGCA

(R) GGCTCTGGGGAAGGGCATTT

**BAX**

(F) CCAAGAAGCTGAGCGAGTGT

(R) CCGGAGGAAGTCCAATGTC

**GRP78**

(F) AAGAACCAGCTCACCTCCAACCC

(R) TTCAACCACCTTGAACGGCAA

**CHOP**

(F) GGAAACAGAGTGGTCATTCCC

(R) CTGCTTGAGCCGTTCATTCTC

**BCL-2**

(F) GAGTTCGCCGAGATGTCCAG

(R) TCACTTGTGGCTCAGATAGG

**IRE1**

(F) CAGCAAGAGGACAGGCTCAA

(R) CAGTGAGGCCGCATAGTCAA
